# Medicine as She Is Learned

**Published:** 1895-10

**Authors:** 


### MEDICINE AS SHE IS LEARNED.

Following are some of the questions and answers gathered from reports of different medical boards:

Q. Give composition of the blood?

A. Composed of red and white blud corpuscles and plasmon.

Q. Describe the functions of the lungs?

A. The lungs is a cavity which holds in inspiration and also holds some expiation.

Q. How many bones in the head?

A. Four, oxcipital, two parental and frontal.

Q. How many elements are there?

A. There is only one element. [Unfortunately, we are not told what it is.]

Q. What is a metal?

A. It is a substance made up of different compositions.

Q. What are antacids?

A. Don't know.

And still there are some people who pretend to believe that there is no use for examining boards.

